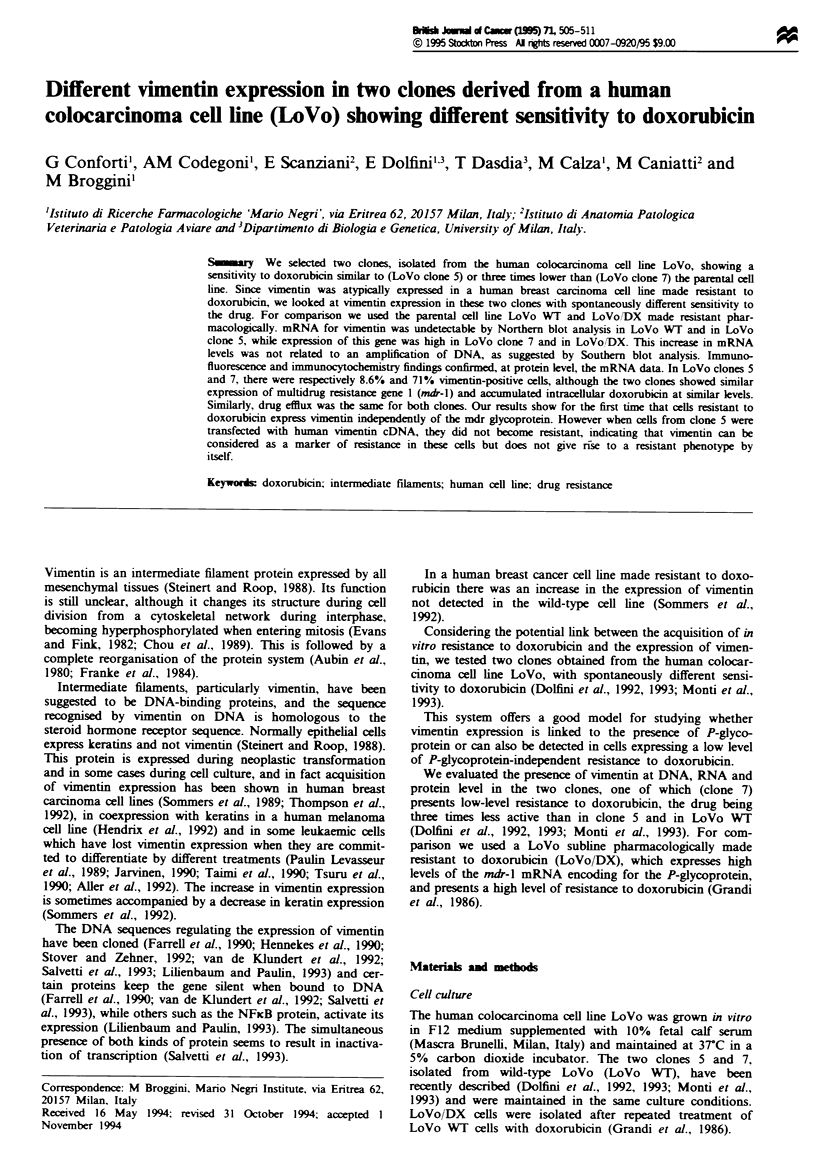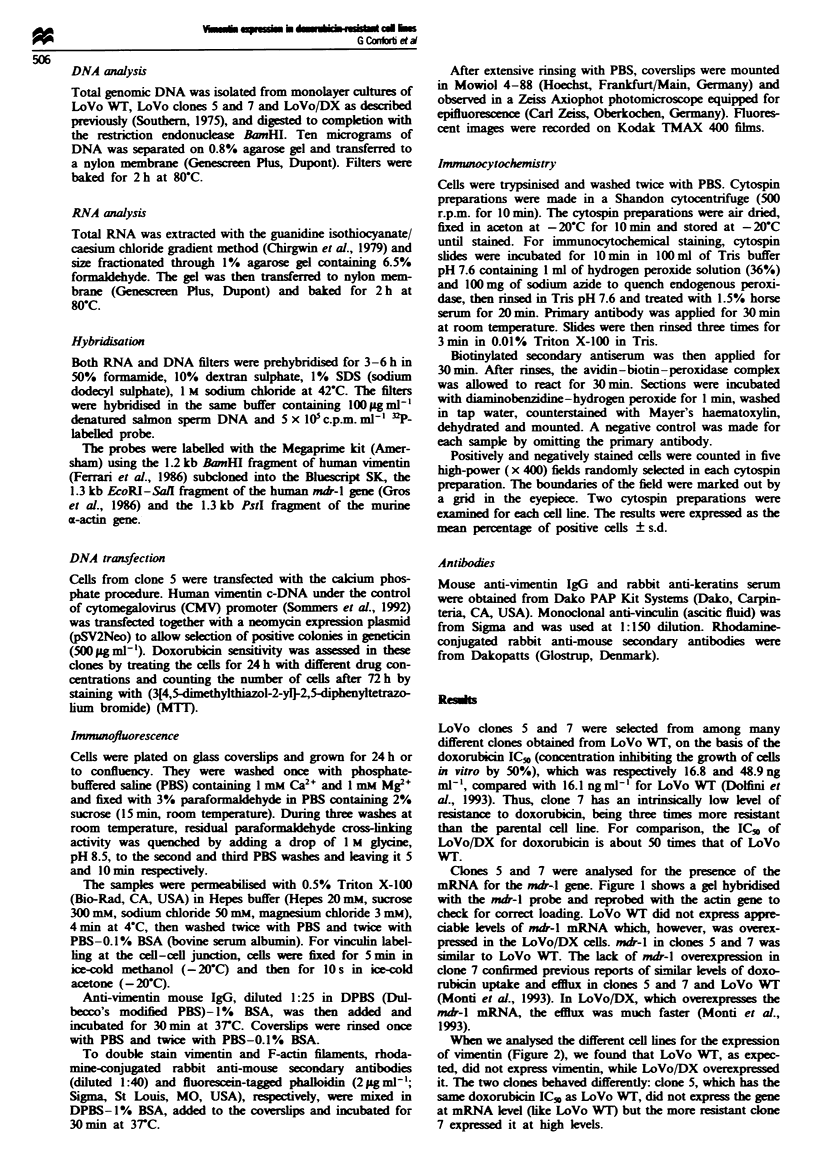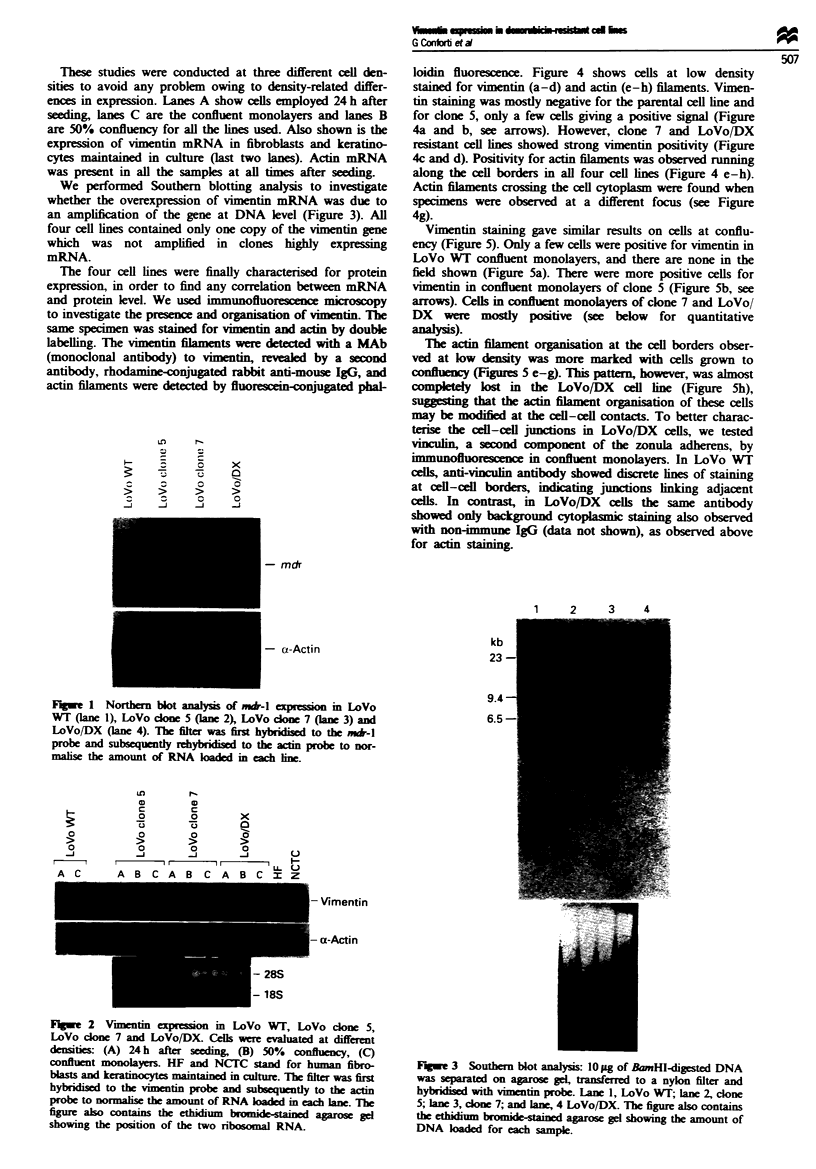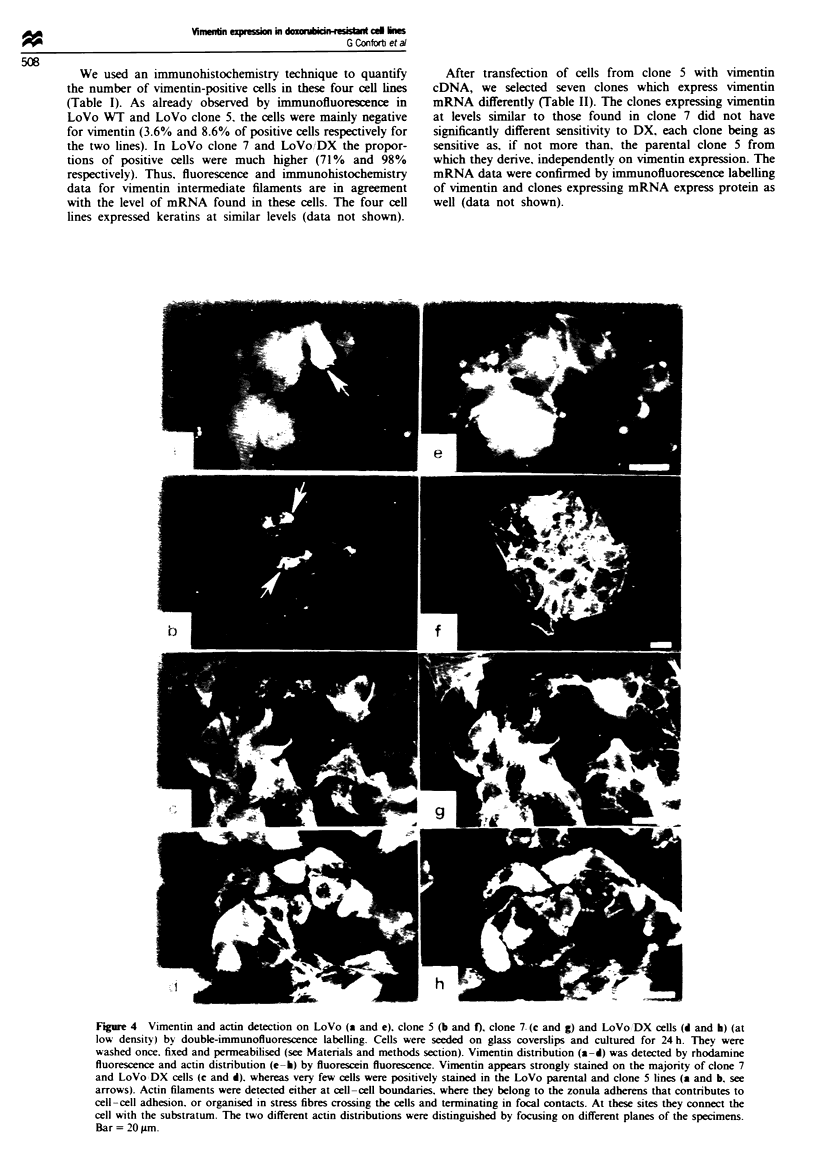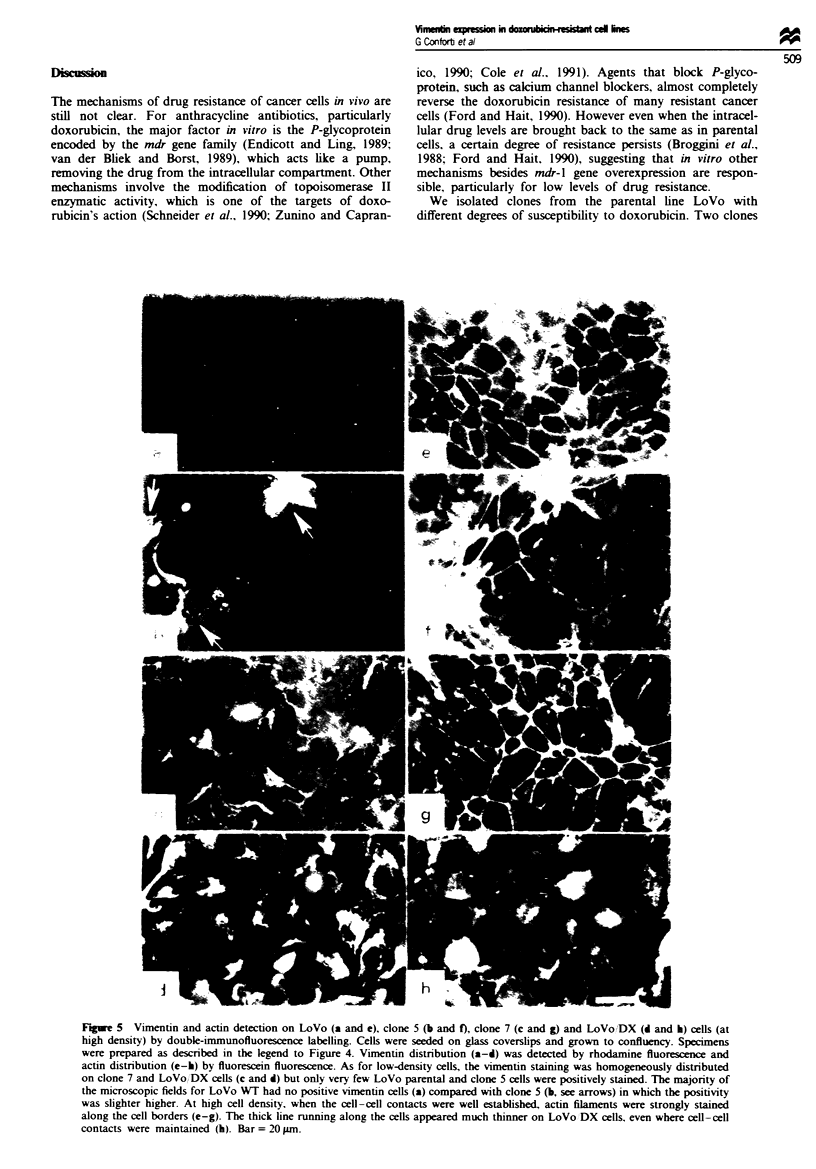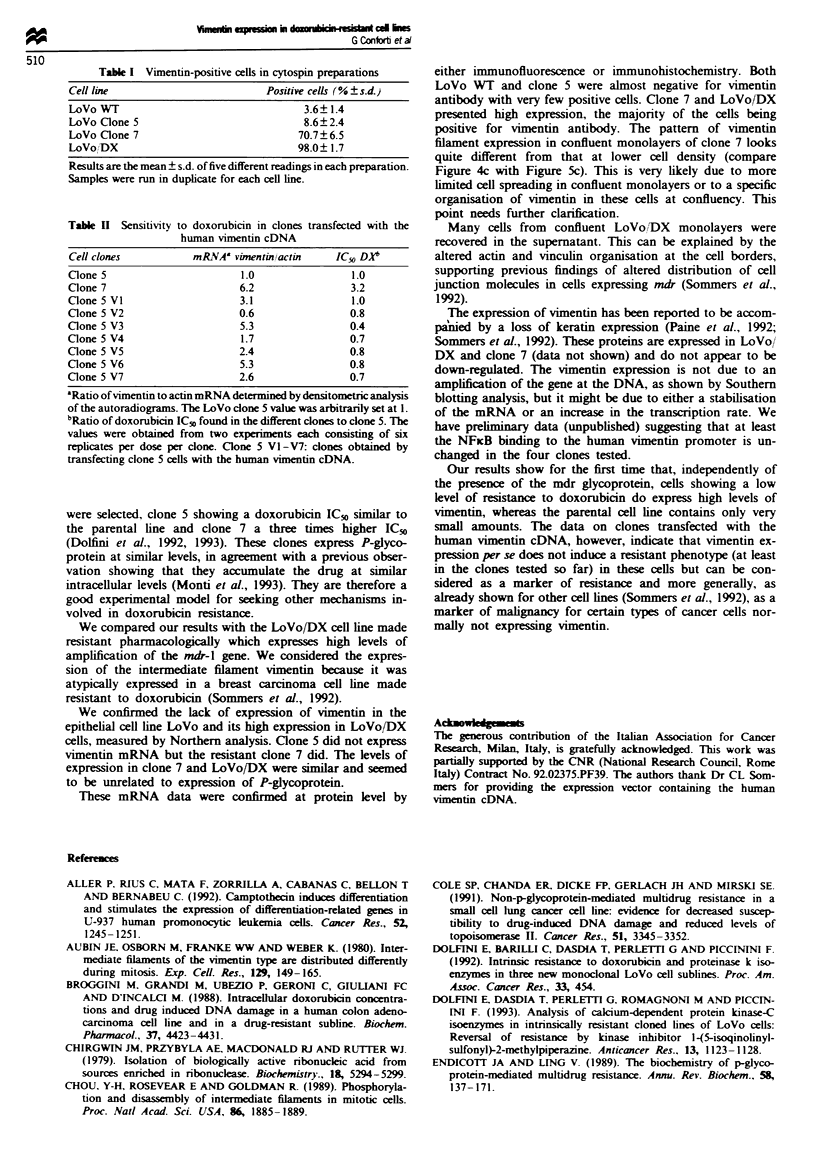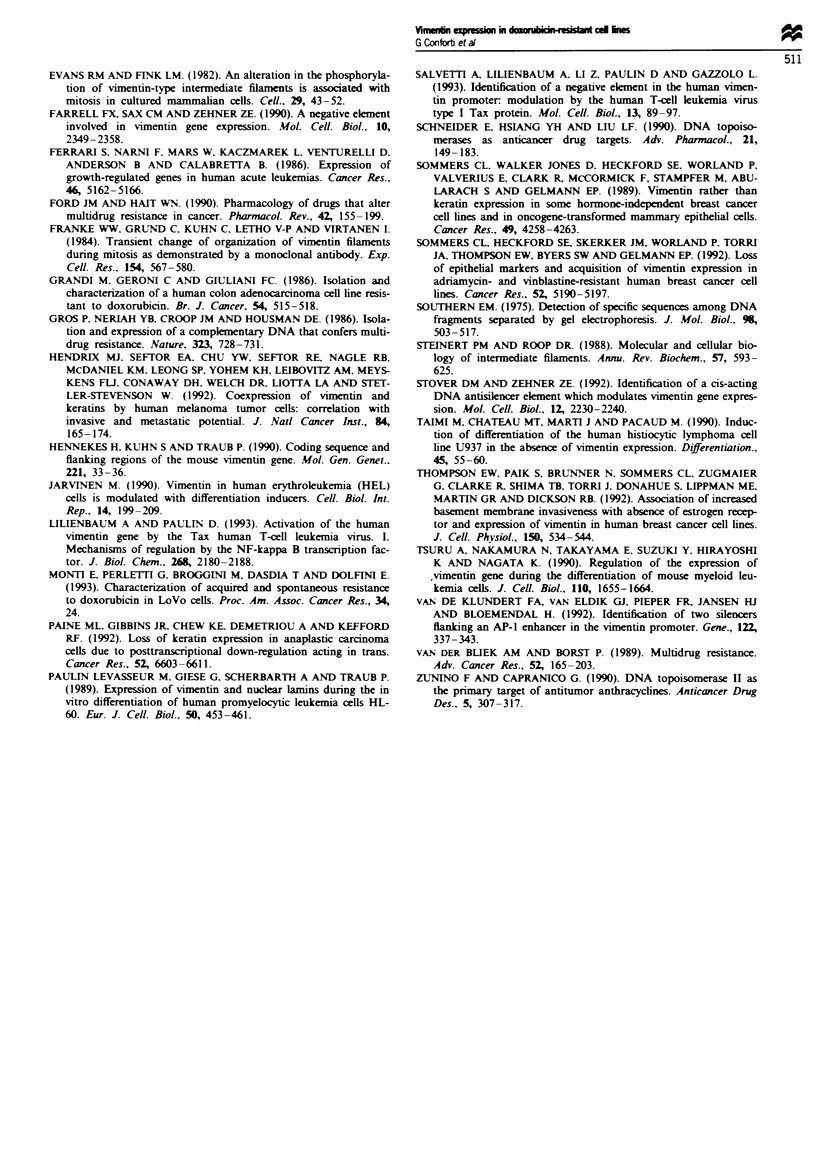# Different vimentin expression in two clones derived from a human colocarcinoma cell line (LoVo) showing different sensitivity to doxorubicin.

**DOI:** 10.1038/bjc.1995.101

**Published:** 1995-03

**Authors:** G. Conforti, A. M. Codegoni, E. Scanziani, E. Dolfini, T. Dasdia, M. Calza, M. Caniatti, M. Broggini

**Affiliations:** Istituto di Ricerche Farmacologiche, Mario Negri, Milan, Italy.

## Abstract

**Images:**


© 1995 Stockton Press All rights reserved 0007-0920/95 $9.00

### Different vimentin expression in two clones derived from a human colocarcinoma cell line (LoVo) showing different sensitivity to doxorubicin

G Conforti[1], AM Codegoni[1], E Scanziani[2], E Dolfini[1,3], T Dasdia[3], M Calza[1], M Caniatti[2] and M Broggini[1]

[1]*Istituto di Ricerche Farmacologiche 'Mario Negri', via Eritrea 62, 20157 Milan, Italy;* [2]*Istituto di Anatomia Patologica Veterinaria e Patologia Aviare and* [3]*Dipartimento di Biologia e Genetica, University of Milan, Italy.*

**Summary** We selected two clones, isolated from the human colocarcinoma cell line LoVo, showing a sensitivity to doxorubicin similar to (LoVo clone 5) or three times lower than (LoVo clone 7) the parental cell line. Since vimentin was atypically expressed in a human breast carcinoma cell line made resistant to doxorubicin, we looked at vimentin expression in these two clones with spontaneously different sensitivity to the drug. For comparison we used the parental cell line LoVo WT and LoVo/DX made resistant pharmacologically. mRNA for vimentin was undetectable by Northern blot analysis in LoVo WT and in LoVo clone 5, while expression of this gene was high in LoVo clone 7 and in LoVo/DX. This increase in mRNA levels was not related to an amplification of DNA, as suggested by Southern blot analysis. Immunofluorescence and immunocytochemistry findings confirmed, at protein level, the mRNA data. In LoVo clones 5 and 7, there were respectively 8.6% and 71% vimentin-positive cells, although the two clones showed similar expression of multidrug resistance gene 1 (*mdr*-1) and accumulated intracellular doxorubicin at similar levels. Similarly, drug efflux was the same for both clones. Our results show for the first time that cells resistant to doxorubicin express vimentin independently of the mdr glycoprotein. However when cells from clone 5 were transfected with human vimentin cDNA, they did not become resistant, indicating that vimentin can be considered as a marker of resistance in these cells but does not give rise to a resistant phenotype by itself.

**Keywords:** doxorubicin; intermediate filaments; human cell line; drug resistance

Vimentin is an intermediate filament protein expressed by all mesenchymal tissues (Steinert and Roop, 1988). Its function is still unclear, although it changes its structure during cell division from a cytoskeletal network during interphase, becoming hyperphosphorylated when entering mitosis (Evans and Fink, 1982; Chou *et al.*, 1989). This is followed by a complete reorganisation of the protein system (Aubin *et al.*, 1980; Franke *et al.*, 1984).

Intermediate filaments, particularly vimentin, have been suggested to be DNA-binding proteins, and the sequence recognised by vimentin on DNA is homologous to the steroid hormone receptor sequence. Normally epithelial cells express keratins and not vimentin (Steinert and Roop, 1988). This protein is expressed during neoplastic transformation and in some cases during cell culture, and in fact acquisition of vimentin expression has been shown in human breast carcinoma cell lines (Sommers *et al.*, 1989; Thompson *et al.*, 1992), in coexpression with keratins in a human melanoma cell line (Hendrix *et al.*, 1992) and in some leukaemic cells which have lost vimentin expression when they are committed to differentiate by different treatments (Paulin Levasseur *et al.*, 1989; Jarvinen, 1990; Taimi *et al.*, 1990; Tsuru *et al.*, 1990; Aller *et al.*, 1992). The increase in vimentin expression is sometimes accompanied by a decrease in keratin expression (Sommers *et al.*, 1992).

The DNA sequences regulating the expression of vimentin have been cloned (Farrell *et al.*, 1990; Hennekes *et al.*, 1990; Stover and Zehner, 1992; van de Klundert *et al.*, 1992; Salvetti *et al.*, 1993; Lilienbaum and Paulin, 1993) and certain proteins keep the gene silent when bound to DNA (Farrell *et al.*, 1990; van de Klundert *et al.*, 1992; Salvetti *et al.*, 1993), while others such as the NFκB protein, activate its expression (Lilienbaum and Paulin, 1993). The simultaneous presence of both kinds of protein seems to result in inactivation of transcription (Salvetti *et al.*, 1993).

Correspondence: M Broggini, Mario Negri Institute, via Eritrea 62, 20157 Milan, Italy
Received 16 May 1994; revised 31 October 1994; accepted 1 November 1994

In a human breast cancer cell line made resistant to doxorubicin there was an increase in the expression of vimentin not detected in the wild-type cell line (Sommers *et al.*, 1992).

Considering the potential link between the acquisition of *in vitro* resistance to doxorubicin and the expression of vimentin, we tested two clones obtained from the human colocarcinoma cell line LoVo, with spontaneously different sensitivity to doxorubicin (Dolfini *et al.*, 1992, 1993; Monti *et al.*, 1993).

This system offers a good model for studying whether vimentin expression is linked to the presence of *P*-glycoprotein or can also be detected in cells expressing a low level of *P*-glycoprotein-independent resistance to doxorubicin.

We evaluated the presence of vimentin at DNA, RNA and protein level in the two clones, one of which (clone 7) presents low-level resistance to doxorubicin, the drug being three times less active than in clone 5 and in LoVo WT (Dolfini *et al.*, 1992, 1993; Monti *et al.*, 1993). For comparison we used a LoVo subline pharmacologically made resistant to doxorubicin (LoVo/DX), which expresses high levels of the *mdr*-1 mRNA encoding for the *P*-glycoprotein, and presents a high level of resistance to doxorubicin (Grandi *et al.*, 1986).

### Materials and methods

### *Cell culture*

The human colocarcinoma cell line LoVo was grown *in vitro* in F12 medium supplemented with 10% fetal calf serum (Mascra Brunelli, Milan, Italy) and maintained at 37°C in a 5% carbon dioxide incubator. The two clones 5 and 7, isolated from wild-type LoVo (LoVo WT), have been recently described (Dolfini *et al.*, 1992, 1993; Monti *et al.*, 1993) and were maintained in the same culture conditions. LoVo/DX cells were isolated after repeated treatment of LoVo WT cells with doxorubicin (Grandi *et al.*, 1986).

*DNA analysis*

Total genomic DNA was isolated from monolayer cultures of LoVo WT, LoVo clones 5 and 7 and LoVo/DX as described previously (Southern, 1975), and digested to completion with the restriction endonuclease *Bam*HI. Ten micrograms of DNA was separated on 0.8% agarose gel and transferred to a nylon membrane (Genescreen Plus, Dupont). Filters were baked for 2 h at 80°C.

*RNA analysis*

Total RNA was extracted with the guanidine isothiocyanate/ caesium chloride gradient method (Chirgwin *et al*., 1979) and size fractionated through 1% agarose gel containing 6.5% formaldehyde. The gel was then transferred to nylon membrane (Genescreen Plus, Dupont) and baked for 2 h at 80°C.

*Hybridisation*

Both RNA and DNA filters were prehybridised for 3–6 h in 50% formamide, 10% dextran sulphate, 1% SDS (sodium dodecyl sulphate), 1 M sodium chloride at 42°C. The filters were hybridised in the same buffer containing 100 μg $ml^{-1}$ denatured salmon sperm DNA and $5 \times 10^5$ c.p.m. $ml^{-1}$ $^{32}P$-labelled probe.

The probes were labelled with the Megaprime kit (Amersham) using the 1.2 kb *Bam*HI fragment of human vimentin (Ferrari *et al*., 1986) subcloned into the Bluescript SK, the 1.3 kb *Eco*RI–*Sal*I fragment of the human *mdr*-1 gene (Gros *et al*., 1986) and the 1.3 kb *Pst*I fragment of the murine α-actin gene.

*DNA transfection*

Cells from clone 5 were transfected with the calcium phosphate procedure. Human vimentin c-DNA under the control of cytomegalovirus (CMV) promoter (Sommers *et al*., 1992) was transfected together with a neomycin expression plasmid (pSV2Neo) to allow selection of positive colonies in geneticin (500 μg $ml^{-1}$). Doxorubicin sensitivity was assessed in these clones by treating the cells for 24 h with different drug concentrations and counting the number of cells after 72 h by staining with (3[4,5-dimethylthiazol-2-yl]-2,5-diphenyltetrazolium bromide) (MTT).

*Immunofluorescence*

Cells were plated on glass coverslips and grown for 24 h or to confluency. They were washed once with phosphate-buffered saline (PBS) containing 1 mM $Ca^{2+}$ and 1 mM $Mg^{2+}$ and fixed with 3% paraformaldehyde in PBS containing 2% sucrose (15 min, room temperature). During three washes at room temperature, residual paraformaldehyde cross-linking activity was quenched by adding a drop of 1 M glycine, pH 8.5, to the second and third PBS washes and leaving it 5 and 10 min respectively.

The samples were permeabilised with 0.5% Triton X-100 (Bio-Rad, CA, USA) in Hepes buffer (Hepes 20 mM, sucrose 300 mM, sodium chloride 50 mM, magnesium chloride 3 mM), 4 min at 4°C, then washed twice with PBS and twice with PBS–0.1% BSA (bovine serum albumin). For vinculin labelling at the cell–cell junction, cells were fixed for 5 min in ice-cold methanol (–20°C) and then for 10 s in ice-cold acetone (–20°C).

Anti-vimentin mouse IgG, diluted 1:25 in DPBS (Dulbecco's modified PBS)–1% BSA, was then added and incubated for 30 min at 37°C. Coverslips were rinsed once with PBS and twice with PBS–0.1% BSA.

To double stain vimentin and F-actin filaments, rhodamine-conjugated rabbit anti-mouse secondary antibodies (diluted 1:40) and fluorescein-tagged phalloidin (2 μg $ml^{-1}$; Sigma, St Louis, MO, USA), respectively, were mixed in DPBS–1% BSA, added to the coverslips and incubated for 30 min at 37°C.

After extensive rinsing with PBS, coverslips were mounted in Mowiol 4–88 (Hoechst, Frankfurt/Main, Germany) and observed in a Zeiss Axiophot photomicroscope equipped for epifluorescence (Carl Zeiss, Oberkochen, Germany). Fluorescent images were recorded on Kodak TMAX 400 films.

*Immunocytochemistry*

Cells were trypsinised and washed twice with PBS. Cytospin preparations were made in a Shandon cytocentrifuge (500 r.p.m. for 10 min). The cytospin preparations were air dried, fixed in aceton at –20°C for 10 min and stored at –20°C until stained. For immunocytochemical staining, cytospin slides were incubated for 10 min in 100 ml of Tris buffer pH 7.6 containing 1 ml of hydrogen peroxide solution (36%) and 100 mg of sodium azide to quench endogenous peroxidase, then rinsed in Tris pH 7.6 and treated with 1.5% horse serum for 20 min. Primary antibody was applied for 30 min at room temperature. Slides were then rinsed three times for 3 min in 0.01% Triton X-100 in Tris.

Biotinylated secondary antiserum was then applied for 30 min. After rinses, the avidin–biotin–peroxidase complex was allowed to react for 30 min. Sections were incubated with diaminobenzidine–hydrogen peroxide for 1 min, washed in tap water, counterstained with Mayer's haematoxylin, dehydrated and mounted. A negative control was made for each sample by omitting the primary antibody.

Positively and negatively stained cells were counted in five high-power (× 400) fields randomly selected in each cytospin preparation. The boundaries of the field were marked out by a grid in the eyepiece. Two cytospin preparations were examined for each cell line. The results were expressed as the mean percentage of positive cells ± s.d.

*Antibodies*

Mouse anti-vimentin IgG and rabbit anti-keratins serum were obtained from Dako PAP Kit Systems (Dako, Carpinteria, CA, USA). Monoclonal anti-vinculin (ascitic fluid) was from Sigma and was used at 1:150 dilution. Rhodamine-conjugated rabbit anti-mouse secondary antibodies were from Dakopatts (Glostrup, Denmark).

### Results

LoVo clones 5 and 7 were selected from among many different clones obtained from LoVo WT, on the basis of the doxorubicin $IC_{50}$ (concentration inhibiting the growth of cells *in vitro* by 50%), which was respectively 16.8 and 48.9 ng $ml^{-1}$, compared with 16.1 ng $ml^{-1}$ for LoVo WT (Dolfini *et al*., 1993). Thus, clone 7 has an intrinsically low level of resistance to doxorubicin, being three times more resistant than the parental cell line. For comparison, the $IC_{50}$ of LoVo/DX for doxorubicin is about 50 times that of LoVo WT.

Clones 5 and 7 were analysed for the presence of the mRNA for the *mdr*-1 gene. Figure 1 shows a gel hybridised with the *mdr*-1 probe and reprobed with the actin gene to check for correct loading. LoVo WT did not express appreciable levels of *mdr*-1 mRNA which, however, was overexpressed in the LoVo/DX cells. *mdr*-1 in clones 5 and 7 was similar to LoVo WT. The lack of *mdr*-1 overexpression in clone 7 confirmed previous reports of similar levels of doxorubicin uptake and efflux in clones 5 and 7 and LoVo WT (Monti *et al*., 1993). In LoVo/DX, which overexpresses the *mdr*-1 mRNA, the efflux was much faster (Monti *et al*., 1993).

When we analysed the different cell lines for the expression of vimentin (Figure 2), we found that LoVo WT, as expected, did not express vimentin, while LoVo/DX overexpressed it. The two clones behaved differently: clone 5, which has the same doxorubicin $IC_{50}$ as LoVo WT, did not express the gene at mRNA level (like LoVo WT) but the more resistant clone 7 expressed it at high levels.

These studies were conducted at three different cell densities to avoid any problem owing to density-related differences in expression. Lanes A show cells employed 24 h after seeding, lanes C are the confluent monolayers and lanes B are 50% confluency for all the lines used. Also shown is the expression of vimentin mRNA in fibroblasts and keratinocytes maintained in culture (last two lanes). Actin mRNA was present in all the samples at all times after seeding.

We performed Southern blotting analysis to investigate whether the overexpression of vimentin mRNA was due to an amplification of the gene at DNA level (Figure 3). All four cell lines contained only one copy of the vimentin gene which was not amplified in clones highly expressing mRNA.

The four cell lines were finally characterised for protein expression, in order to find any correlation between mRNA and protein level. We used immunofluorescence microscopy to investigate the presence and organisation of vimentin. The same specimen was stained for vimentin and actin by double labelling. The vimentin filaments were detected with a MAb (monoclonal antibody) to vimentin, revealed by a second antibody, rhodamine-conjugated rabbit anti-mouse IgG, and actin filaments were detected by fluorescein-conjugated phalloidin fluorescence. Figure 4 shows cells at low density stained for vimentin (a–d) and actin (e–h) filaments. Vimentin staining was mostly negative for the parental cell line and for clone 5, only a few cells giving a positive signal (Figure 4a and b, see arrows). However, clone 7 and LoVo/DX resistant cell lines showed strong vimentin positivity (Figure 4c and d). Positivity for actin filaments was observed running along the cell borders in all four cell lines (Figure 4 e–h). Actin filaments crossing the cell cytoplasm were found when specimens were observed at a different focus (see Figure 4g).

Vimentin staining gave similar results on cells at confluency (Figure 5). Only a few cells were positive for vimentin in LoVo WT confluent monolayers, and there are none in the field shown (Figure 5a). There were more positive cells for vimentin in confluent monolayers of clone 5 (Figure 5b, see arrows). Cells in confluent monolayers of clone 7 and LoVo/DX were mostly positive (see below for quantitative analysis).

The actin filament organisation at the cell borders observed at low density was more marked with cells grown to confluency (Figures 5 e–g). This pattern, however, was almost completely lost in the LoVo/DX cell line (Figure 5h), suggesting that the actin filament organisation of these cells may be modified at the cell–cell contacts. To better characterise the cell–cell junctions in LoVo/DX cells, we tested vinculin, a second component of the zonula adherens, by immunofluorescence in confluent monolayers. In LoVo WT cells, anti-vinculin antibody showed discrete lines of staining at cell–cell borders, indicating junctions linking adjacent cells. In contrast, in LoVo/DX cells the same antibody showed only background cytoplasmic staining also observed with non-immune IgG (data not shown), as observed above for actin staining.

**Figure 1** Northern blot analysis of *mdr*-1 expression in LoVo WT (lane 1), LoVo clone 5 (lane 2), LoVo clone 7 (lane 3) and LoVo/DX (lane 4). The filter was first hybridised to the *mdr*-1 probe and subsequently rehybridised to the actin probe to normalise the amount of RNA loaded in each line.

**Figure 2** Vimentin expression in LoVo WT, LoVo clone 5, LoVo clone 7 and LoVo/DX. Cells were evaluated at different densities: (A) 24 h after seeding, (B) 50% confluency, (C) confluent monolayers. HF and NCTC stand for human fibroblasts and keratinocytes maintained in culture. The filter was first hybridised to the vimentin probe and subsequently to the actin probe to normalise the amount of RNA loaded in each lane. The figure also contains the ethidium bromide-stained agarose gel showing the position of the two ribosomal RNA.

**Figure 3** Southern blot analysis: 10 μg of *Bam*HI-digested DNA was separated on agarose gel, transferred to a nylon filter and hybridised with vimentin probe. Lane 1, LoVo WT; lane 2, clone 5; lane 3, clone 7; and lane, 4 LoVo/DX. The figure also contains the ethidium bromide-stained agarose gel showing the amount of DNA loaded for each sample.

We used an immunohistochemistry technique to quantify the number of vimentin-positive cells in these four cell lines (Table I). As already observed by immunofluorescence in LoVo WT and LoVo clone 5, the cells were mainly negative for vimentin (3.6% and 8.6% of positive cells respectively for the two lines). In LoVo clone 7 and LoVo/DX the proportions of positive cells were much higher (71% and 98% respectively). Thus, fluorescence and immunohistochemistry data for vimentin intermediate filaments are in agreement with the level of mRNA found in these cells. The four cell lines expressed keratins at similar levels (data not shown).

After transfection of cells from clone 5 with vimentin cDNA, we selected seven clones which express vimentin mRNA differently (Table II). The clones expressing vimentin at levels similar to those found in clone 7 did not have significantly different sensitivity to DX, each clone being as sensitive as, if not more than, the parental clone 5 from which they derive, independently on vimentin expression. The mRNA data were confirmed by immunofluorescence labelling of vimentin and clones expressing mRNA express protein as well (data not shown).

**Figure 4** Vimentin and actin detection on LoVo (**a** and **e**), clone 5 (**b** and **f**), clone 7 (**c** and **g**) and LoVo/DX cells (**d** and **h**) (at low density) by double-immunofluorescence labelling. Cells were seeded on glass coverslips and cultured for 24 h. They were washed once, fixed and permeabilised (see Materials and methods section). Vimentin distribution (**a**–**d**) was detected by rhodamine fluorescence and actin distribution (**e**–**h**) by fluorescein fluorescence. Vimentin appears strongly stained on the majority of clone 7 and LoVo/DX cells (**c** and **d**), whereas very few cells were positively stained in the LoVo parental and clone 5 lines (**a** and **b**, see arrows). Actin filaments were detected either at cell–cell boundaries, where they belong to the zonula adherens that contributes to cell–cell adhesion, or organised in stress fibres crossing the cells and terminating in focal contacts. At these sites they connect the cell with the substratum. The two different actin distributions were distinguished by focusing on different planes of the specimens. Bar = 20 μm.

### Discussion

The mechanisms of drug resistance of cancer cells *in vivo* are still not clear. For anthracycline antibiotics, particularly doxorubicin, the major factor *in vitro* is the *P*-glycoprotein encoded by the *mdr* gene family (Endicott and Ling, 1989; van der Bliek and Borst, 1989), which acts like a pump, removing the drug from the intracellular compartment. Other mechanisms involve the modification of topoisomerase II enzymatic activity, which is one of the targets of doxorubicin's action (Schneider *et al.*, 1990; Zunino and Capranico, 1990; Cole *et al.*, 1991). Agents that block *P*-glycoprotein, such as calcium channel blockers, almost completely reverse the doxorubicin resistance of many resistant cancer cells (Ford and Hait, 1990). However even when the intracellular drug levels are brought back to the same as in parental cells, a certain degree of resistance persists (Broggini *et al.*, 1988; Ford and Hait, 1990), suggesting that *in vitro* other mechanisms besides *mdr*-1 gene overexpression are responsible, particularly for low levels of drug resistance.

We isolated clones from the parental line LoVo with different degrees of susceptibility to doxorubicin. Two clones

**Figure 5** Vimentin and actin detection on LoVo (**a** and **e**), clone 5 (**b** and **f**), clone 7 (**c** and **g**) and LoVo/DX (**d** and **h**) cells (at high density) by double-immunofluorescence labelling. Cells were seeded on glass coverslips and grown to confluency. Specimens were prepared as described in the legend to Figure 4. Vimentin distribution (**a**–**d**) was detected by rhodamine fluorescence and actin distribution (**e**–**h**) by fluorescein fluorescence. As for low-density cells, the vimentin staining was homogeneously distributed on clone 7 and LoVo/DX cells (**c** and **d**) but only very few LoVo parental and clone 5 cells were positively stained. The majority of the microscopic fields for LoVo WT had no positive vimentin cells (**a**) compared with clone 5 (**b**, see arrows) in which the positivity was slighter higher. At high cell density, when the cell–cell contacts were well established, actin filaments were strongly stained along the cell borders (**e**–**g**). The thick line running along the cells appeared much thinner on LoVo/DX cells, even where cell–cell contacts were maintained (**h**). Bar = 20 $\mu$m.

Table I Vimentin-positive cells in cytospin preparations

| Cell line | Positive cells (% ± s.d.) |
|---|---|
| LoVo WT | 3.6 ± 1.4 |
| LoVo Clone 5 | 8.6 ± 2.4 |
| LoVo Clone 7 | 70.7 ± 6.5 |
| LoVo/DX | 98.0 ± 1.7 |

Results are the mean ± s.d. of five different readings in each preparation. Samples were run in duplicate for each cell line.

Table II Sensitivity to doxorubicin in clones transfected with the human vimentin cDNA

| Cell clones | mRNA[a] vimentin/actin | $IC_{50}$ DX[b] |
|---|---|---|
| Clone 5 | 1.0 | 1.0 |
| Clone 7 | 6.2 | 3.2 |
| Clone 5 V1 | 3.1 | 1.0 |
| Clone 5 V2 | 0.6 | 0.8 |
| Clone 5 V3 | 5.3 | 0.4 |
| Clone 5 V4 | 1.7 | 0.7 |
| Clone 5 V5 | 2.4 | 0.8 |
| Clone 5 V6 | 5.3 | 0.8 |
| Clone 5 V7 | 2.6 | 0.7 |

[a]Ratio of vimentin to actin mRNA determined by densitometric analysis of the autoradiograms. The LoVo clone 5 value was arbitrarily set at 1. [b]Ratio of doxorubicin $IC_{50}$ found in the different clones to clone 5. The values were obtained from two experiments each consisting of six replicates per dose per clone. Clone 5 V1–V7: clones obtained by transfecting clone 5 cells with the human vimentin cDNA.

were selected, clone 5 showing a doxorubicin $IC_{50}$ similar to the parental line and clone 7 a three times higher $IC_{50}$ (Dolfini *et al.*, 1992, 1993). These clones express *P*-glycoprotein at similar levels, in agreement with a previous observation showing that they accumulate the drug at similar intracellular levels (Monti *et al.*, 1993). They are therefore a good experimental model for seeking other mechanisms involved in doxorubicin resistance.

We compared our results with the LoVo/DX cell line made resistant pharmacologically which expresses high levels of amplification of the *mdr*-1 gene. We considered the expression of the intermediate filament vimentin because it was atypically expressed in a breast carcinoma cell line made resistant to doxorubicin (Sommers *et al.*, 1992).

We confirmed the lack of expression of vimentin in the epithelial cell line LoVo and its high expression in LoVo/DX cells, measured by Northern analysis. Clone 5 did not express vimentin mRNA but the resistant clone 7 did. The levels of expression in clone 7 and LoVo/DX were similar and seemed to be unrelated to expression of *P*-glycoprotein.

These mRNA data were confirmed at protein level by either immunofluorescence or immunohistochemistry. Both LoVo WT and clone 5 were almost negative for vimentin antibody with very few positive cells. Clone 7 and LoVo/DX presented high expression, the majority of the cells being positive for vimentin antibody. The pattern of vimentin filament expression in confluent monolayers of clone 7 looks quite different from that at lower cell density (compare Figure 4c with Figure 5c). This is very likely due to more limited cell spreading in confluent monolayers or to a specific organisation of vimentin in these cells at confluency. This point needs further clarification.

Many cells from confluent LoVo/DX monolayers were recovered in the supernatant. This can be explained by the altered actin and vinculin organisation at the cell borders, supporting previous findings of altered distribution of cell junction molecules in cells expressing *mdr* (Sommers *et al.*, 1992).

The expression of vimentin has been reported to be accompanied by a loss of keratin expression (Paine *et al.*, 1992; Sommers *et al.*, 1992). These proteins are expressed in LoVo/DX and clone 7 (data not shown) and do not appear to be down-regulated. The vimentin expression is not due to an amplification of the gene at the DNA, as shown by Southern blotting analysis, but it might be due to either a stabilisation of the mRNA or an increase in the transcription rate. We have preliminary data (unpublished) suggesting that at least the NFκB binding to the human vimentin promoter is unchanged in the four clones tested.

Our results show for the first time that, independently of the presence of the mdr glycoprotein, cells showing a low level of resistance to doxorubicin do express high levels of vimentin, whereas the parental cell line contains only very small amounts. The data on clones transfected with the human vimentin cDNA, however, indicate that vimentin expression *per se* does not induce a resistant phenotype (at least in the clones tested so far) in these cells but can be considered as a marker of resistance and more generally, as already shown for other cell lines (Sommers *et al.*, 1992), as a marker of malignancy for certain types of cancer cells normally not expressing vimentin.

**Acknowledgements**
The generous contribution of the Italian Association for Cancer Research, Milan, Italy, is gratefully acknowledged. This work was partially supported by the CNR (National Research Council, Rome Italy) Contract No. 92.02375.PF39. The authors thank Dr CL Sommers for providing the expression vector containing the human vimentin cDNA.

### References

ALLER P, RIUS C, MATA F, ZORRILLA A, CABANAS C, BELLON T AND BERNABEU C. (1992). Camptothecin induces differentiation and stimulates the expression of differentiation-related genes in U-937 human promonocytic leukemia cells. *Cancer Res.*, **52**, 1245–1251.

AUBIN JE, OSBORN M, FRANKE WW AND WEBER K. (1980). Intermediate filaments of the vimentin type are distributed differently during mitosis. *Exp. Cell. Res.*, **129**, 149–165.

BROGGINI M, GRANDI M, UBEZIO P, GERONI C, GIULIANI FC AND D'INCALCI M. (1988). Intracellular doxorubicin concentrations and drug induced DNA damage in a human colon adenocarcinoma cell line and in a drug-resistant subline. *Biochem. Pharmacol.*, **37**, 4423–4431.

CHIRGWIN JM, PRZYBYLA AE, MACDONALD RJ AND RUTTER WJ. (1979). Isolation of biologically active ribonucleic acid from sources enriched in ribonuclease. *Biochemistry.*, **18**, 5294–5299.

CHOU, Y-H, ROSEVEAR E AND GOLDMAN R. (1989). Phosphorylation and disassembly of intermediate filaments in mitotic cells. *Proc. Natl Acad. Sci. USA*, **86**, 1885–1889.

COLE SP, CHANDA ER, DICKE FP, GERLACH JH AND MIRSKI SE. (1991). Non-p-glycoprotein-mediated multidrug resistance in a small cell lung cancer cell line: evidence for decreased susceptibility to drug-induced DNA damage and reduced levels of topoisomerase II. *Cancer Res.*, **51**, 3345–3352.

DOLFINI E, BARILLI C, DASDIA T, PERLETTI G AND PICCININI F. (1992). Intrinsic resistance to doxorubicin and proteinase k isoenzymes in three new monoclonal LoVo cell sublines. *Proc. Am. Assoc. Cancer Res.*, **33**, 454.

DOLFINI E, DASDIA T, PERLETTI G, ROMAGNONI M AND PICCININI F. (1993). Analysis of calcium-dependent protein kinase-C isoenzymes in intrinsically resistant cloned lines of LoVo cells: Reversal of resistance by kinase inhibitor 1-(5-isoqinolinylsulfonyl)-2-methylpiperazine. *Anticancer Res.*, **13**, 1123–1128.

ENDICOTT JA AND LING V. (1989). The biochemistry of p-glycoprotein-mediated multidrug resistance. *Annu. Rev. Biochem.*, **58**, 137–171.

EVANS RM AND FINK LM. (1982). An alteration in the phosphorylation of vimentin-type intermediate filaments is associated with mitosis in cultured mammalian cells. *Cell.*, **29**, 43-52.

FARRELL FX, SAX CM AND ZEHNER ZE. (1990). A negative element involved in vimentin gene expression. *Mol. Cell. Biol.*, **10**, 2349-2358.

FERRARI S, NARNI F, MARS W, KACZMAREK L, VENTURELLI D, ANDERSON B AND CALABRETTA B. (1986). Expression of growth-regulated genes in human acute leukemias. *Cancer Res.*, **46**, 5162-5166.

FORD JM AND HAIT WN. (1990). Pharmacology of drugs that alter multidrug resistance in cancer. *Pharmacol. Rev.*, **42**, 155-199.

FRANKE WW, GRUND C, KUHN C, LETHO V-P AND VIRTANEN I. (1984). Transient change of organization of vimentin filaments during mitosis as demonstrated by a monoclonal antibody. *Exp. Cell. Res.*, **154**, 567-580.

GRANDI M, GERONI C AND GIULIANI FC. (1986). Isolation and characterization of a human colon adenocarcinoma cell line resistant to doxorubicin. *Br. J. Cancer*, **54**, 515-518.

GROS P, NERIAH YB, CROOP JM AND HOUSMAN DE. (1986). Isolation and expression of a complementary DNA that confers multidrug resistance. *Nature*, **323**, 728-731.

HENDRIX MJ, SEFTOR EA, CHU YW, SEFTOR RE, NAGLE RB, McDANIEL KM, LEONG SP, YOHEM KH, LEIBOVITZ AM, MEYSKENS FLJ, CONAWAY DH, WELCH DR, LIOTTA LA AND STETLER-STEVENSON W. (1992). Coexpression of vimentin and keratins by human melanoma tumor cells: correlation with invasive and metastatic potential. *J. Natl Cancer Inst.*, **84**, 165-174.

HENNEKES H, KUHN S AND TRAUB P. (1990). Coding sequence and flanking regions of the mouse vimentin gene. *Mol. Gen. Genet.*, **221**, 33-36.

JARVINEN M. (1990). Vimentin in human erythroleukemia (HEL) cells is modulated with differentiation inducers. *Cell. Biol. Int. Rep.*, **14**, 199-209.

LILIENBAUM A AND PAULIN D. (1993). Activation of the human vimentin gene by the Tax human T-cell leukemia virus. I. Mechanisms of regulation by the NF-kappa B transcription factor. *J. Biol. Chem.*, **268**, 2180-2188.

MONTI E, PERLETTI G, BROGGINI M, DASDIA T AND DOLFINI E. (1993). Characterization of acquired and spontaneous resistance to doxorubicin in LoVo cells. *Proc. Am. Assoc. Cancer Res.*, **34**, 24.

PAINE ML, GIBBINS JR, CHEW KE, DEMETRIOU A AND KEFFORD RF. (1992). Loss of keratin expression in anaplastic carcinoma cells due to posttranscriptional down-regulation acting in trans. *Cancer Res.*, **52**, 6603-6611.

PAULIN LEVASSEUR M, GIESE G, SCHERBARTH A AND TRAUB P. (1989). Expression of vimentin and nuclear lamins during the in vitro differentiation of human promyelocytic leukemia cells HL-60. *Eur. J. Cell. Biol.*, **50**, 453-461.

SALVETTI A, LILIENBAUM A, LI Z, PAULIN D AND GAZZOLO L. (1993). Identification of a negative element in the human vimentin promoter: modulation by the human T-cell leukemia virus type I Tax protein. *Mol. Cell. Biol.*, **13**, 89-97.

SCHNEIDER E, HSIANG YH AND LIU LF. (1990). DNA topoisomerases as anticancer drug targets. *Adv. Pharmacol.*, **21**, 149-183.

SOMMERS CL, WALKER JONES D, HECKFORD SE, WORLAND P, VALVERIUS E, CLARK R, McCORMICK F, STAMPFER M, ABULARACH S AND GELMANN EP. (1989). Vimentin rather than keratin expression in some hormone-independent breast cancer cell lines and in oncogene-transformed mammary epithelial cells. *Cancer Res.*, **49**, 4258-4263.

SOMMERS CL, HECKFORD SE, SKERKER JM, WORLAND P, TORRI JA, THOMPSON EW, BYERS SW AND GELMANN EP. (1992). Loss of epithelial markers and acquisition of vimentin expression in adriamycin- and vinblastine-resistant human breast cancer cell lines. *Cancer Res.*, **52**, 5190-5197.

SOUTHERN EM. (1975). Detection of specific sequences among DNA fragments separated by gel electrophoresis. *J. Mol. Biol.*, **98**, 503-517.

STEINERT PM AND ROOP DR. (1988). Molecular and cellular biology of intermediate filaments. *Annu. Rev. Biochem.*, **57**, 593-625.

STOVER DM AND ZEHNER ZE. (1992). Identification of a cis-acting DNA antisilencer element which modulates vimentin gene expression. *Mol. Cell. Biol.*, **12**, 2230-2240.

TAIMI M, CHATEAU MT, MARTI J AND PACAUD M. (1990). Induction of differentiation of the human histiocytic lymphoma cell line U937 in the absence of vimentin expression. *Differentiation.*, **45**, 55-60.

THOMPSON EW, PAIK S, BRUNNER N, SOMMERS CL, ZUGMAIER G, CLARKE R, SHIMA TB, TORRI J, DONAHUE S, LIPPMAN ME, MARTIN GR AND DICKSON RB. (1992). Association of increased basement membrane invasiveness with absence of estrogen receptor and expression of vimentin in human breast cancer cell lines. *J. Cell. Physiol.*, **150**, 534-544.

TSURU A, NAKAMURA N, TAKAYAMA E, SUZUKI Y, HIRAYOSHI K AND NAGATA K. (1990). Regulation of the expression of vimentin gene during the differentiation of mouse myeloid leukemia cells. *J. Cell. Biol.*, **110**, 1655-1664.

VAN DE KLUNDERT FA, VAN ELDIK GJ, PIEPER FR, JANSEN HJ AND BLOEMENDAL H. (1992). Identification of two silencers flanking an AP-1 enhancer in the vimentin promoter. *Gene.*, **122**, 337-343.

VAN DER BLIEK AM AND BORST P. (1989). Multidrug resistance. *Adv. Cancer Res.*, **52**, 165-203.

ZUNINO F AND CAPRANICO G. (1990). DNA topoisomerase II as the primary target of antitumor anthracyclines. *Anticancer Drug Des.*, **5**, 307-317.